# Zosteriform Lichen Planus in a Young Woman: A Case Report of the Diagnostic Challenges and Psychodermatologic Associations

**DOI:** 10.7759/cureus.113131

**Published:** 2026-07-21

**Authors:** Leiby Orellana Banegas, Luz M Lemus, Steffany O Villeda, Leslie N Crosdaile, Aimeé Gomez Sanzio, Jose E Benitez, Raul Urbina

**Affiliations:** 1 General Medicine, Universidad Católica de Honduras, San Pedro Sula, HND; 2 General Medicine, Universidad Católica de Honduras, Copán, HND; 3 General Medicine, Universidad Católica de Honduras “Nuestra Señora Reina de la Paz”, San Pedro Sula, HND; 4 Medicine, Universidad Católica de Honduras “Nuestra Señora Reina de la Paz”, San Pedro Sula, HND; 5 Internal Medicine, Universidad Católica de Honduras, San Pedro Sula, HND

**Keywords:** dermatomal, lichen planus, psychodermatology, psychological stress, psychoneuroimmunology, topical corticosteroids, zosteriform lichen planus

## Abstract

Lichen planus (LP) is a chronic T-cell-mediated inflammatory dermatosis affecting the skin, mucous membranes, nails, and scalp. Zosteriform lichen planus (ZLP) is a rare clinical variant characterized by a unilateral dermatomal distribution that closely mimics herpes zoster, creating a significant diagnostic challenge.

We report the case of a 22-year-old woman who presented with pruritic, hyperpigmented papules distributed along the left L3 dermatome. Because histopathologic confirmation was unavailable, the diagnosis was established clinically by the treating dermatologist based on the characteristic lesion morphology, dermatomal distribution, chronic-relapsing course, therapeutic response, and exclusion of the principal differential diagnoses. Following dermatologic evaluation, the patient underwent formal psychological assessment by a licensed psychologist using the 10-item Perceived Stress Scale (PSS-10), which demonstrated moderate perceived stress (15/40), predominantly related to academic and personal life stressors. Treatment with topical clobetasol propionate 0.05% resulted in marked clinical improvement, while subsequent recurrences remained temporally associated with periods of increased perceived stress and responded to reintroduction of topical corticosteroid therapy together with stress-management strategies.

This case highlights the diagnostic challenges of clinically diagnosed ZLP in a resource-limited setting and demonstrates the importance of systematic clinical reasoning when biopsy is not feasible, while reaffirming that histopathologic confirmation remains the preferred method whenever available. It also supports formal psychosocial assessment as a valuable adjunct to the multidisciplinary management of selected patients with recurrent, stress-associated inflammatory skin disease.

## Introduction

Lichen planus (LP) is a chronic, immune-mediated inflammatory dermatosis affecting the skin, mucous membranes, nails, and scalp [1]. The global prevalence of cutaneous LP is estimated to range from approximately 0.2% to 1% of the adult population, with oral involvement reported in up to 1%-4% of the population [2]; the disease shows a slight female predominance (approximately 1.5:1) [2] and a peak incidence between the third and sixth decades of life [2]. The disease is driven by a cytotoxic CD8+ T-cell response directed against basal keratinocytes at the dermal-epidermal junction, resulting in the characteristic lichenoid interface dermatitis that defines the disease histopathologically [3]. Clinically, LP typically presents as flat-topped, polygonal, violaceous papules with fine white reticulate markings (Wickham's striae), most commonly involving the flexural surfaces of the wrists, dorsal hands, lower back, and ankles [2].

Cutaneous LP encompasses multiple morphological variants, including classic, hypertrophic, linear, annular, bullous, ulcerative, pigmented, and zosteriform subtypes [4]. Among these, zosteriform lichen planus (ZLP) is considered one of the rarest clinical variants [5,6]. Unlike Blaschko-linear LP, which follows the lines of Blaschko and is thought to reflect cutaneous mosaicism, ZLP typically follows a unilateral dermatomal distribution that closely mimics herpes zoster [6]. Importantly, ZLP is not preceded by a vesicular eruption and is not associated with varicella-zoster virus (VZV) reactivation [6]. Recent reports have expanded the recognized clinical spectrum of ZLP, including cases following chemotherapy [7], after recombinant zoster vaccination [8], and as isolated spontaneous eruptions with no antecedent herpes zoster [5,6], highlighting both its rarity and the diversity of its clinical presentations. Despite this increasing recognition, the ZLP literature consists largely of isolated case reports, and clinicopathologic characterization of the entity remains scarce.

An expanding body of psychodermatology literature supports a bidirectional relationship between psychological stress and inflammatory skin disease, mediated through the brain-skin axis [9]. Psychological stress activates the hypothalamic-pituitary-adrenal (HPA) axis and the sympathetic nervous system, leading to the release of cortisol, catecholamines, substance P (SP), and calcitonin gene-related peptide (CGRP) [10,11]. SP is a potent activator of cutaneous mast cells, promoting degranulation and neurogenic inflammation, whereas CGRP has been shown to modulate antigen presentation by epidermal Langerhans cells and downstream T-cell responses. These neuroimmune mechanisms have been described in psoriasis and atopic dermatitis [10], and psychological stress has similarly been associated with disease activity in LP, particularly oral LP [12], although evidence specifically involving cutaneous ZLP remains scarce.

We present the case of a 22-year-old woman with clinically diagnosed ZLP involving the left L3 dermatome. The case demonstrated a temporal association between recurrent disease activity and moderate perceived stress, as measured by the Perceived Stress Scale (PSS-10) [13]. This case adds to the limited body of literature by describing ZLP in a young woman from Central America and highlights the diagnostic challenges encountered in a resource-limited setting where histopathologic confirmation was not feasible. It further emphasizes the value of transparent clinical reasoning, careful exclusion of relevant differential diagnoses, and multidisciplinary assessment, including standardized psychological evaluation, in selected patients with recurrent disease activity.

## Case presentation

A 22-year-old woman with no prior personal or family history of dermatologic or autoimmune disease presented in October 2019 with a several-week history of mildly pruritic, hyperpigmented papules on the medial aspect of the left leg. She reported no preceding trauma, infection, vaccination, new medication use, or systemic symptoms and denied any previous episode of herpes zoster or chronic inflammatory skin disease. The patient reported that lesion onset coincided with a prolonged period of academic stress accompanied by concurrent personal life stressors, which she perceived as temporally associated with the appearance of her skin lesions.

The initial lesion was a solitary, non-painful, non-scaling hyperpigmented papule of approximately 2 cm on the medial left leg. Over the following weeks, new lesions appeared in a progressive, linear dermatomal distribution along the medial left thigh and leg, strictly confined to the left side without crossing the midline. No satellite lesions, vesicles, or crusting were noted. By January 2020, the number of lesions had increased markedly, with confluent hyperpigmentation across the affected dermatome (Figure 1).

**Figure 1:**
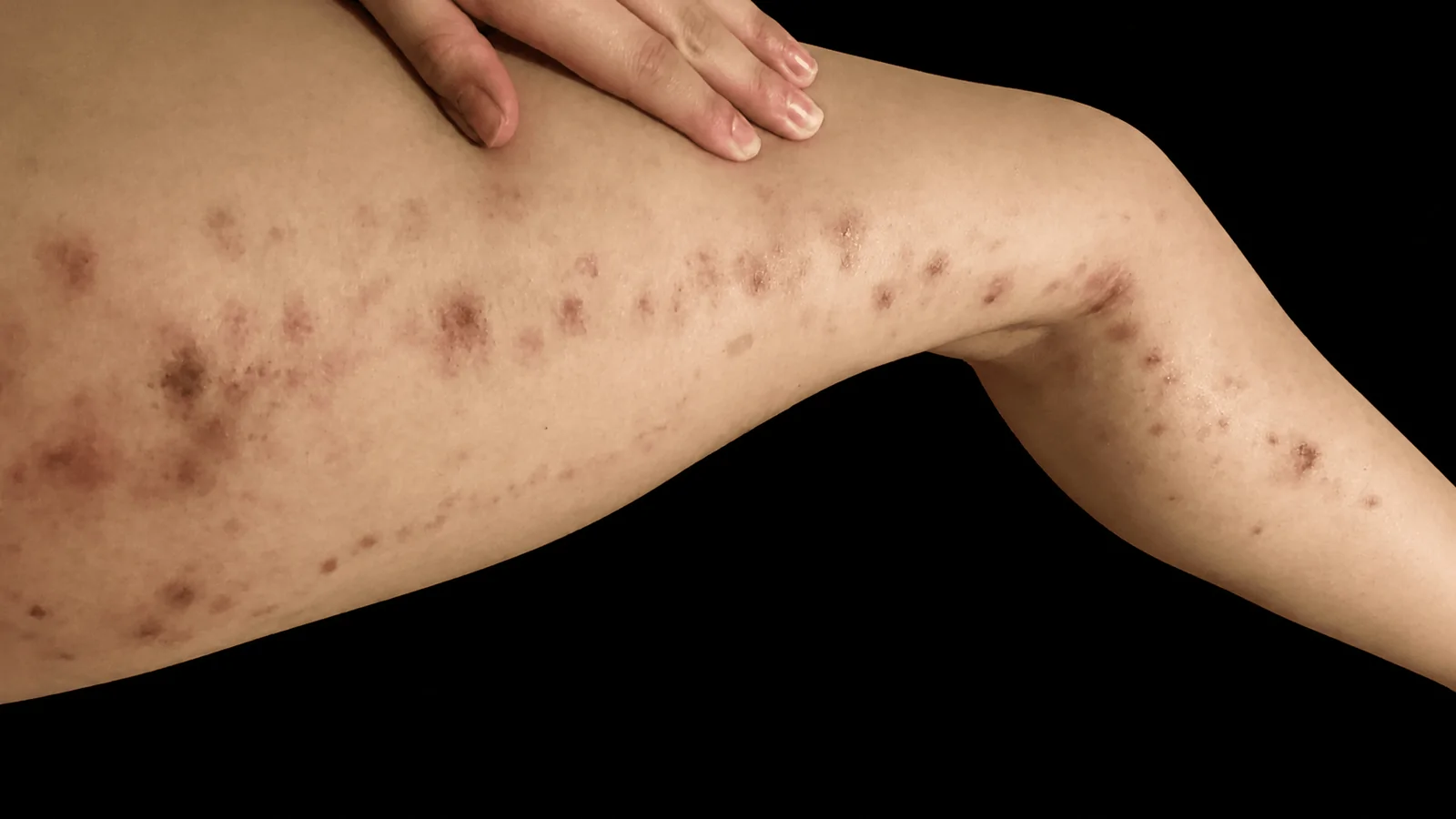
Active phase of zosteriform lichen planus (January 2020) Multiple erythematous to hyperpigmented, slightly elevated papules distributed in a strictly unilateral dermatomal pattern along the medial aspect of the left thigh and leg, consistent with the L3 dermatome. Note the linear arrangement of lesions without midline crossing and the absence of vesicles or crusting, features that distinguish this presentation from herpes zoster.

Physical examination revealed multiple slightly elevated, hyperpigmented, non-scaling papules distributed unilaterally along the medial aspect of the left thigh and leg in a dermatomal pattern consistent with the L3 distribution. No Wickham's striae were identified on clinical examination. There were no lesions on the mucous membranes, nails, scalp, or contralateral limb. No lymphadenopathy or hepatosplenomegaly was noted.

Laboratory evaluation, including complete blood count, metabolic panel, thyroid function tests, hormonal panel, and allergen-specific IgE screening, was unremarkable. Viral serology for VZV and herpes simplex virus (HSV) was negative, making active herpetic infection unlikely in the context of the overall clinical presentation. Histopathologic confirmation was not obtained because the lesions had already begun to resolve at the time of specialist evaluation and because skin biopsy was not readily available at the treating institution due to local resource limitations. Although histopathology is the preferred method for confirming atypical presentations of LP, the treating dermatologist considered the characteristic morphology, unilateral dermatomal distribution, chronic relapsing course, therapeutic response, and exclusion of the principal differential diagnoses sufficient to support a clinical diagnosis of unilateral ZLP.

Following dermatologic evaluation, the patient underwent an independent, formal psychological assessment conducted by a licensed psychologist. Perceived stress was assessed using the validated 10-item PSS-10, yielding a score of 15/40, consistent with moderate perceived stress. The principal stressors identified were ongoing academic demands and concurrent personal life events. No primary psychiatric disorder was diagnosed. Stress-management strategies were discussed as part of the patient's multidisciplinary care plan.

Treatment was initiated with topical clobetasol propionate 0.05% ointment applied once daily. Oral loratadine 10 mg once daily was prescribed intermittently for symptomatic pruritus. After three months of consistent therapy, significant resolution of active papules was achieved, with residual post-inflammatory hyperpigmentation (PIH) managed with a topical depigmenting agent (Figure 2). The active phase of the disease lasted approximately six months. The clinical course progressed from an initial solitary lesion through progressive dermatomal spread to clinical diagnosis, treatment with topical clobetasol propionate, and eventual resolution with residual PIH. Long-term follow-up showed episodic stress-associated recurrences that responded favorably to reintroduction of topical corticosteroid therapy (Table 1).

**Figure 2:**
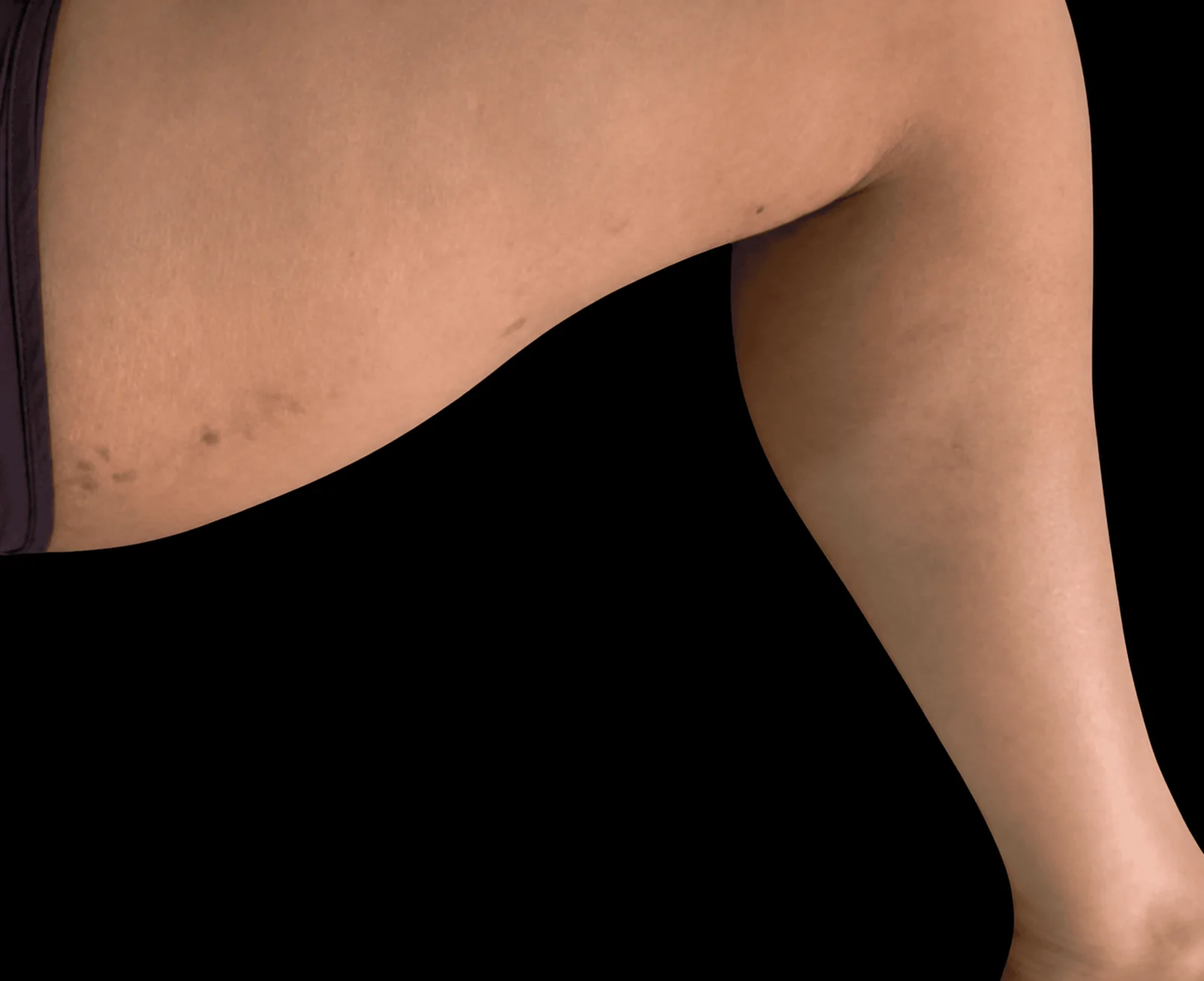
Post-treatment clinical response (May 2020) Marked resolution of active papules following three months of topical clobetasol propionate 0.05% once daily. Residual post-inflammatory hyperpigmentation (PIH) is visible along the previously affected dermatomal distribution, with no active erythematous or raised lesions. The unilateral distribution and absence of new lesion formation are consistent with treatment response.

**Table 1:** Clinical course and management timeline PIH, post-inflammatory hyperpigmentation; LP, lichen planus; PRN, as needed

Date	Event	Findings	Management
Oct 2019	Disease Onset	Solitary hyperpigmented papule on the medial left leg	Observation
Nov-Dec 2019	Progressive Spread	Development of additional papules in unilateral dermatomal distribution	Emollients and monitoring
Jan 2020	Diagnostic Evaluation	Increased lesion burden and confluent hyperpigmentation	Clinical diagnosis of zosteriform LP
Feb 2020	Psychological Evaluation	PSS-10 score: 15/40 (moderate perceived stress)	Formal assessment by a licensed psychologist; stress-management strategies discussed
Feb-Apr 2020	Active Treatment	Persistent papules and pruritus	Clobetasol propionate 0.05% daily; loratadine PRN
May-Oct 2020	Resolution Phase	Resolution of active lesions with residual PIH	Topical depigmenting therapy
Long-Term Follow-Up	Recurrence Management	Stress-associated episodic flares	Reintroduction of topical corticosteroids

Follow-up over the subsequent months demonstrated a favorable clinical course, with rare episodic recurrences. Each relapse was temporally associated with identifiable periods of heightened psychological stress and was not preceded by infectious prodrome or identifiable contact exposure. Recurrences responded promptly to the reintroduction of topical clobetasol propionate, without requiring systemic therapy. During follow-up, a formal psychological assessment documented moderate perceived stress. The patient reported that disease recurrences consistently occurred during periods of increased academic and personal stress and improved following reintroduction of topical corticosteroid therapy, alongside adherence to stress-management strategies.

## Discussion

Diagnostic challenges

Only a limited number of cases of ZLP have been reported in the literature [5,6]. The majority of reported cases have involved middle-aged or older women with single- or multiple-dermatome involvement, most commonly in the thoracic or lumbar regions [5,6]; for example, a 39-year-old woman with truncal ZLP [5] and a 62-year-old woman with chemotherapy-induced ZLP following mastectomy [7]. Reports specifically in young adult women are exceptionally rare; the closest comparable case in the literature is a 27-year-old woman with linear ZLP pigmentosus, a related but histopathologically distinct entity confirmed by biopsy [14]. Our case is therefore particularly noteworthy for its atypical demographic presentation in a young woman, its confinement to a single lower-extremity dermatome (L3), and the consistent temporal association between psychological stress and disease onset and recurrence, a combination not previously described together in this variant.

Stress as a trigger

The pathogenesis of LP centers on a CD8+ cytotoxic T-lymphocyte (CTL) response directed against basal keratinocytes expressing altered surface antigens, resulting in lichenoid interface dermatitis with vacuolar alteration and Civatte body formation [3]. In the zosteriform variant, the mechanism underlying dermatomal restriction of this autoimmune response remains incompletely understood. Two principal hypotheses have been proposed: an isomorphic response (Koebner phenomenon) triggered by subclinical nerve injury or mechanical trauma along the affected dermatome; and a neurogenic mechanism in which cutaneous nerve fibers within a specific dermatome release neuroimmunological mediators that prime the local immune environment for lichenoid inflammation [6,9].

The second hypothesis is directly relevant to the role of psychological stress in our patient. Psychological stress activates the HPA axis and the sympathetic adrenomedullary system, increasing systemic levels of cortisol and catecholamines. Paradoxically, chronic stress leads to relative glucocorticoid resistance in peripheral tissues despite elevated circulating cortisol, thereby favoring a net pro-inflammatory state [9,11]. Concurrently, stress stimulates cutaneous sensory nerve terminals to release neuropeptides, particularly SP, CGRP, and nerve growth factor (NGF), which promote mast cell degranulation, dendritic cell activation, and amplification of the CD8+ T-cell cytotoxic response [10,11]. This neurogenic inflammatory circuit, operating along specific dermatomes richly innervated by nociceptive fibers, may contribute to the dermatomal restriction of LP in psychologically stressed individuals.

Academic stress in particular is a well-recognized and highly prevalent stressor in young adults: a 2022 survey of 843 college students found academic stress to be a leading correlate of impaired mental well-being across this population [15]. This supports the biological plausibility of academic stress as the principal precipitant identified in our patient's PSS-10 assessment. This aligns with evidence linking psychological stress, anxiety, and depression to disease activity in LP, particularly oral LP [12]. This relationship may plausibly extend to the cutaneous zosteriform variant described here, though we emphasize that this is, at most, a temporal association observed in a single case rather than an established causal mechanism.

Differential diagnosis

The differential diagnosis of ZLP includes entities that can produce unilateral, dermatomal, or linear cutaneous eruptions (Table 2). The most clinically relevant mimicker is herpes zoster, with which ZLP shares its dermatomal distribution but differs fundamentally in its lack of prodromal neuralgia, absence of vesicular or crusting lesions, negative VZV serology, and chronic relapsing, rather than self-limited monophasic, course. Linear psoriasis and linear LP following Blaschko's lines must also be considered, as must contact dermatitis with a linear pattern and, in younger patients, incontinentia pigmenti in its later stages. The clinical diagnosis of ZLP in this patient was supported by multiple converging findings despite the absence of histopathological confirmation (Table 3).

**Table 2:** Differential diagnosis of unilateral dermatomal eruptions LP, lichen planus; VZV, varicella-zoster virus; NF1, neurofibromatosis type 1

Condition	Distribution	Typical Findings	Key Distinguishing Features
Herpes Zoster	Dermatomal	Vesicles and crusts	Neuralgia, VZV positivity, self-limited course
Zosteriform LP	Dermatomal	Hyperpigmented or violaceous papules	Chronic-relapsing, no vesicles, stress-associated
Linear LP	Blaschko lines	Violaceous papules	Non-dermatomal pattern
Linear Psoriasis	Segmental/Blaschkoid	Erythematous plaques with silvery scale	Auspitz sign, nail involvement
Contact Dermatitis	Exposure-related	Eczematous lesions	Positive exposure history
Segmental Neurofibromatosis	Segmental	Café-au-lait macules, neurofibromas	NF1 mosaicism

**Table 3:** Clinical features supporting the diagnosis of zosteriform lichen planus in the absence of histopathological confirmation LP, lichen planus; VZV, varicella-zoster virus; HSV, herpes simplex virus

Diagnostic Feature	Findings in This Case
Clinical morphology consistent with LP	Flat-topped, polygonal, hyperpigmented pruritic papules; no vesicles, no crusting
Unilateral dermatomal distribution	Strictly left-sided, L3 dermatomal pattern; no midline crossing
Absence of vesicular or herpetiform lesions	No blisters, no crusts, no prodromal neuralgia at any point during the clinical course
Negative viral serology (VZV, HSV)	VZV IgM/IgG negative; HSV IgM negative; no viral reactivation detected
Chronic-relapsing (non-monophasic) course	Multiple flares over >12 months; not self-limited; recurred with psychological stress
Response to topical high-potency corticosteroids	Significant resolution after 3 months of clobetasol propionate 0.05%; repeated response on reintroduction
Temporal association with psychological stress	Patient-reported consistent temporal relationship; academic/personal stress preceded each flare
Absence of alternative diagnoses after exclusion workup	Herpes zoster, linear LP, lichen striatus, and cutaneous metastasis were excluded clinically and serologically

Therapeutic response

Topical high-potency corticosteroids, particularly clobetasol propionate 0.05%, constitute the accepted first-line therapy for localized LP [1]. Their mechanism involves suppression of T-cell activation, reduction of pro-inflammatory cytokine release (IL-2, IFN-γ, and TNF-α), and inhibition of keratinocyte apoptosis at the basal layer. In our patient, a three-month course yielded significant resolution of active papules, consistent with established response rates for localized LP. The lack of need for systemic immunosuppression, including systemic corticosteroids, acitretin, ciclosporin, or hydroxychloroquine, further supports the utility of topical therapy in geographically limited disease [1,2].

Limitations

The absence of histopathological confirmation is the principal limitation of this report. Nevertheless, the unilateral dermatomal distribution, absence of vesicular lesions, negative viral studies, chronic-relapsing course, and response to topical corticosteroids collectively support the clinical diagnosis. Dermoscopic characterization, which in LP typically reveals Wickham's striae, whitish reticular structures, and dotted or linear vessels on a violaceous background, was also not documented. Beyond these diagnostic gaps, this report has several additional limitations. It is based on a single case, which precludes generalization. It also relies on patient-reported temporal association between stress and disease activity, which is inherently subject to recall and attribution bias, even when anchored by a validated instrument such as the PSS-10. Finally, complementary psychometric or quality-of-life instruments, such as the Dermatology Life Quality Index (DLQI), were not administered, which would have allowed a more complete characterization of disease burden alongside perceived stress. Formal psychiatric evaluation against DSM-5 criteria was not performed, and no primary psychiatric disorder was diagnosed; whether a codeable stress-related diagnosis (e.g., adjustment disorder) was present cannot be determined from the data collected, and we did not attempt to assign one retrospectively. Future cases of suspected ZLP should include biopsy and dermoscopy whenever feasible, particularly given the rarity of this variant and the importance of excluding histological mimickers, together with structured psychometric assessment where a stress association is suspected.

Clinical implications

The psychodermatological dimension of this case has practical clinical implications. A growing body of literature indicates that dermatological patients with comorbid psychological stress or psychiatric illness experience more frequent relapses, poorer treatment adherence, and lower health-related quality of life [12]. This case illustrates this principle in clinical practice: formal psychosocial screening using the PSS-10 identified moderate perceived stress that correlated with both disease onset and subsequent recurrences, and engagement with stress-management strategies accompanied clinical improvement. We suggest that formal psychosocial screening using validated instruments such as the DLQI and PSS-10, with referral to mental health services where indicated, should be considered a valuable adjunct to multidisciplinary care for stress-sensitive dermatoses, including LP. This case supports the potential value of integrating standardized psychosocial assessment into the multidisciplinary management of selected patients with stress-associated inflammatory dermatoses. Prospective studies incorporating histopathologic confirmation and standardized psychometric assessment are needed to better define the relationship between psychological stress and ZLP.

## Conclusions

ZLP presenting in a young woman represents an uncommon demographic variation of an already rare LP variant. This case illustrates the diagnostic challenge posed by unilateral dermatomal eruptions that clinically mimic herpes zoster, particularly when histopathological confirmation is unavailable. The convergence of characteristic clinical morphology, strictly dermatomal distribution, negative viral studies, chronic-relapsing course, and consistent response to topical clobetasol propionate collectively supported the clinical diagnosis. These findings highlight the importance of maintaining a broad differential diagnosis when evaluating unilateral dermatomal eruptions and recognizing ZLP as an uncommon but important diagnostic consideration, particularly in settings where access to dermatopathology is limited. This case also emphasizes the importance of systematic clinical reasoning in resource-limited settings, where histopathologic confirmation may not always be feasible. Careful integration of lesion morphology, dermatomal distribution, exclusion of relevant differential diagnoses, longitudinal follow-up, and response to therapy can support a well-reasoned clinical diagnosis while transparently acknowledging the inherent diagnostic limitations. Although histopathologic confirmation remains the preferred method for confirming atypical presentations of LP, this case illustrates that a structured and transparent clinical approach can assist clinicians in making informed diagnostic and management decisions when biopsy is not feasible. The repeated and temporally consistent association between moderate perceived stress, documented through formal psychological assessment (PSS-10), and disease flares represents the principal distinguishing feature of this report. This pattern is consistent with emerging evidence suggesting that psychoneuroimmunological mechanisms may contribute to disease activity in inflammatory skin disorders. Incorporating psychosocial assessment into dermatologic evaluation may facilitate earlier recognition of potential triggers, improve long-term disease control, and support a more comprehensive, patient-centered approach to care. By documenting this rare presentation, we expand the limited literature on ZLP and encourage further investigation into the role of psychological stress in its pathogenesis and recurrence. Beyond describing a rare dermatologic presentation, this case provides a practical framework for approaching suspected ZLP in settings where histopathologic confirmation cannot be readily obtained while reinforcing that biopsy remains the preferred diagnostic method whenever feasible.

From the patient's perspective, periods of academic and personal stress consistently preceded disease flares. She described the lesions as cosmetically distressing and associated with persistent itching that affected her daily comfort. Following treatment with topical clobetasol, she experienced substantial improvement and reported increased confidence and reduced concern regarding the appearance of the affected area. She continued to engage with the stress-management strategies introduced during her initial psychological assessment and subjectively described improved coping with stress at subsequent follow-up visits; however, perceived stress was not formally re-quantified with a repeat PSS-10, and no additional psychiatric referral was pursued beyond the initial evaluation. She recognized stress-management strategies as an important component of reducing future disease recurrences.
